# Who is at the table and who has the power? Case study analysis of decision-making processes for the Global Financing Facility in Tanzania

**DOI:** 10.1080/16549716.2025.2552531

**Published:** 2025-09-05

**Authors:** Donat Shamba, Jitihada Baraka, Mary V. Kinney, Asha S. George, Georgina Msemo, Joy E. Lawn, Rosie Steege

**Affiliations:** a Department of Health Systems Impact Evaluation and Policy, Ifakara Health Institute, Dar es Salaam, Tanzania; b Department of Infectious Disease Epidemiology and International Health, London School of Hygiene & Tropical Medicine, London, UK; c School of Public Health, University of the Western Cape, Bellville, South Africa; d Global Financing Facility, Department of Health, Nutrition and Population, Dar Es Salaam, Tanzania; e Department of International Public Health, Liverpool School of Tropical Medicine, Liverpool, UK

**Keywords:** Global Financing Facility for Women, Children and Adolescents: Examining National Priorities, Processes and Investments, Tanzania, global health initiative, maternal, newborn, stillbirths, financing policy

## Abstract

**Background:**

In 2015, Tanzania joined the Global Financing Facility (GFF), a global health initiative for Reproductive, Maternal, Newborn, Child, and Adolescent Health and Nutrition (RMNCAH-N). Despite its resource mobilization goals, little is known about power dynamics in GFF policy processes. This paper presents the first power analysis of Tanzania’s GFF engagement.

**Objective:**

To examine policy processes in developing GFF documents during its first two phases in Tanzania.

**Methods:**

An exploratory qualitative case study using document reviews (*n* = 22) and key informant interviews (*n* = 21) conducted in 2022–2023. Data were thematically analyzed and interpreted using Gaventa’s power cube (levels, spaces, and forms of power).

**Results:**

Stakeholders praised the GFF’s country-led, evidence-based approach and local autonomy. However, closed-door decision-making in phase one excluded civil society and the private sector. Invisible power imbalances in funding allocations left stillbirths and adolescent health without dedicated budgets, while vulnerable groups (e.g. people with disabilities) were overlooked. Disbursement-linked indicators emphasized measurable outcomes, reflecting visible power. Phase two showed adaptive learning, with improved inclusivity.

**Conclusion:**

While government-led, global actors (e.g. World Bank, donors) heavily influenced decisions. Greater civil society engagement is needed for accountability. Future efforts must address power imbalances through meaningful citizen participation to strengthen RMNCAH-N services.

## Paper in Context

**Main findings:**

The Global Financing Facility policy process in Tanzania drew from the existing national government strategy and was perceived as country-owned by national stakeholders, but closed-door decision-making and limited transparency excluded civil society and reduced accountability to citizens.

**Added knowledge:**

This is the first power analysis of the Global Financing Facility process in Tanzania, applying the Gaventa Power Cube to reveal how visible, hidden, and invisible power shaped participation, priority-setting, and stakeholder inclusion over time, offering lessons for other global health initiatives.

**Global health impact for policy and action:**

Global health initiatives should be reflexive and intentionally create early, meaningful opportunities for engagement with citizens, including underserved groups, to strengthen accountability and ensure inclusive decision-making.

## Background

The Global Financing Facility (GFF) was launched in July 2015 as an innovative financing mechanism focused on reproductive, maternal, newborn, child, and adolescent health and nutrition (RMNCAH-N) [1,2]. The GFF, hosted by the World Bank, acts as a multi-donor trust fund to catalyse investments [3]. The GFF supports governments to convene partners around a country-led investment case (IC) that prioritizes maternal, newborn, and child health, aligning financial resources from government, development, and other donors and involving stakeholders, including civil society organizations, the private sector, and multilateral institutions [2]. The health financing workplan of the GFF is embedded within the Project Appraisal Document (PAD), which is a World Bank document that describes a specific project financed primarily by the International Bank for Reconstruction and Development (IBRD) and International Development Association (IDA) [4].

Country grants from the GFF are intended to leverage larger loans from the World Bank [5]. Every $1 of GFF donor grant money invested in Tanzania, aims to enable around $4 worth of credit through the World Bank’s International Development Association for the same purpose [2]. Along with catalysing investments, the GFF aims to streamline reporting, strengthen accountability, and support governments in increasing domestic resources over time [6]. The GFF partnership involves governments assuming leadership roles in setting policy agendas and formulating technically sound and financially appropriate strategies and plans. The governance of the GFF, however, provides substantial decision-making power to the World Bank and donors through its ‘investors group,’ which mobilizes funding [2].

Power can be defined as ‘the degree of control over material, human, intellectual, and financial resources exercised by different sections of society’ [7]. It is dynamic, relational, and exercised daily through social practices [8]. Exercises of power are central to shaping resource mobilization, health policies and outcomes [9]. Multiple actors, with their values and positions, must decide on priorities contained within plans and budgets [10], with consequences for health-service users [11]. Power is exerted as an actor’s ability to influence resource allocation as well as their ability to subvert control [12,13]. Therefore, understanding power is critical to understanding funding and priority-setting processes and is integral to improving health outcomes [14–16]. The GFF has the potential to leverage its convening power for impact; however, the power dynamics in the GFF’s policy processes require exploration to support accountability and inform future implementation phases.

Tanzania was among the first four ‘front-runner’ countries supported by the GFF in round one of GFF funding in 2015–2021. Tanzania was purposely selected by the GFF because the government focused on maternal, newborn, and child health with a championship at the presidential level [5,17]. In the second round (2022–2027), the GFF provided funding to strengthen essential health services [18]. This study aims to explore the power processes involved in shaping the development of GFF documents (Investment cases and Government-World Bank Project appraisal Document) in Tanzania over time (between the GFF first and second rounds).

## Methods

### Study design

As part of the Countdown 2030 GFF policy analysis collaboration [19,20], we conducted an exploratory, qualitative case study of GFF policy processes in Tanzania between 2015 and 2022. The multi-disciplinary consortium applied an analytical framework for the case study adapted from the Walt and Gilson health policy triangle, considering context, content, actors, process, and their interactions [19,21], to allow comparison across four African settings [20].

### Study setting

Tanzania met the Millennium Development Goal (MDG) for child survival by 2015 but had slow progress in the reduction of maternal and newborn mortality and low rates of family planning uptake [22]. The national Reproductive, Maternal, Newborn, Child, and Adolescent Health and Nutrition (RMNCAH-N) focussed ‘One Plan’ strategy included targets aligned to the SDGs to address these remaining challenges for reduction of maternal, newborn and under-five mortality [23]. Tanzania has a well-established and functional sector-wide approach (SWAp) mechanism for government-led health financing, enabling development partners to pool resources into a common health basket fund under the government’s common policy framework. The World Bank is a major contributor to health basket funds. Table 1 presents an overview of the relevant priority national targets and status.

**Table 1.** Tanzania’s national targets for mortality or impact of relevance to GFF, showing the current status.

Indicator	Target type	Target level (year)	Status in rate/ratio*	Number of deaths in 2022
***Mortality or outcome targets***				
*Maternal mortality ratio*	*SDG 3.1 #* *TZ HSSP and One Plan III*	*75 per 100,000 births* *(2030)*	*104 per 100,000*	*2,300*
*Stillbirth rate*	*Every Newborn, UNSG planTZ One Plan III*	*12 per 1000 births* *(2030)*	*19 per 1000*	*40,000*
*Neonatal mortality*	*SDG 3.2* *Every Newborn*,*TZ HSSP and OnePlan III*	*12 per 1000 births* *(2030)*	*24 per 1000*	*50,000*
*Under five mortality*	*SDG 3.2TZ HSSP and OnePlan III*	*25 per 1000 births* *(2030)*	*43 per 1000*	*43,000* *after neonatal period*
*Teenage (15–19) pregnancy*	*SDG 3.7TZ HSSP and OnePlan III*	* <20% of girls aged 15–19 years pregnant or have born a child* *(2030)*	*22%*	*N/A*

*Based on (TDHS 2022–2023)

### Study population and selection process

Study participants were purposefully sampled based on their involvement in GFF processes, and snow balling sampling technique was also used to identify further informants. Attention was paid to the diversity of roles, organizations, and gender of respondents. Informants included officials from the Ministry of Health (MoH), Ministry of Finance (MoF), Donor and Development Partners, Implementation Partners, World Bank, GFF, and Civil Society Organization (CSOs). Participants were purposefully sampled based on their involvement in GFF processes. Inclusion criteria included individuals with direct involvement in the GFF IC or PAD development or implementation processes between 2015 and 2022. An initial list of study participants were identified by the researcher JB in consultation with the GFF country liaison focal person (GM), then were contacted by phone and letters to explain the study objectives, and the informed consent process.

To identify the documents, a desk review was conducted to identify and collate relevant documents for analysis of the GFF process in Tanzania. The search covered 2015–2023 and included national health policies, GFF planning documents, academic articles, reports, and grey literature from government and partner webpages and documents from key informants. Documents were screened for relevance to RMNCAH-N priorities, the GFF process, or health sector financing in Tanzania.

### Study tools

Data collection tools were co-designed with the GFF policy analysis collaboration, including a key informant interview guide and a document review extraction template [20], and then adapted for the Tanzanian context.

### Data collection

Data were collected between November 2022 and April 2023 and included a document review and key informant interviews. The document review included 22 related policy documents, strategies, grey literature, and peer-reviewed publications (Table 2).

**Table 2.** List of Tanzanian documents reviewed for policy context.

Name of document	Duration	The government/Agency designed policy
Countdown to 2015: Country case studies	1990–2015	Global Financing facility
Health Sector Strategic Plan (HSSP II)	2005–2010	Ministry of Health
Sharpened One Plan	2008–2015	Ministry of Health
Health Sector Strategic Plan (HSSP III)	2010–2015	Ministry of Health
National Plan for Reproductive, Maternal, Newborn, Child and Adolescent Health & Nutrition (One Plan I)	2011–2015	Ministry of Health
Public Expenditure Review (PER) on Tanzania’s Health Sector	2013–2017	Ministry of Health
Policy brief: Women and Children First: Countdown to Ending Preventable Maternal, Newborn, and Child Deaths in Tanzania	2014	Global Financing facility
Program appraisal document I	2015	World Bank
The GFF’s contribution to domestic resources mobilization for health and nutrition	2015	Global Financing facility
The Global Financing Facility in Tanzania: A Brief Summary	2015–2020	Global Financing facility
Health Sector Strategic Plan (HSSPIV)	2015–2020	Ministry of Health
Mid-Term Review (MTR) of the Health Sector Strategic Plan IV (HSSP IV)	2015–2020	Ministry of Health
Analytical report on the Health Sector Strategic Plan IV (HSSP IV)	2015–2018	Ministry of Health
National Plan for Reproductive, Maternal, Newborn, Child and Adolescent Health & Nutrition (One Plan II)	2016–2020	Ministry of Health
The National Road Map Strategic Plan to Improve Reproductive, Maternal, Newborn, Child & Adolescent Health in Tanzania	2016–2020	Ministry of Health
Every Woman Every Child Global Strategy for Women’s, Children’s, and Adolescents’ Health	2016–2030	WHO/UNICEF
Implementation Plan for the Civil Society Engagement Strategy (CSES) of the Global Financing Facility (GFF)	2017	Global Financing facility
Report about the Global Financing Facility (GFF), detailing its impact on women’s, children’s, and adolescents’ health and nutrition	2018	Global Financing facility
Health Sector Strategic Plan (HSSP V)	2020–2025	Ministry of Health
National Plan for Reproductive, Maternal, Newborn, Child and Adolescent Health & Nutrition (One Plan III)	2022–2026	Ministry of Health
Tanzania Maternal and Child Health Investment Program-Program Appraisal Document. Tanzania – Program-for-Results (IDA)	2022	World Bank
Program appraisal document II	2022	World Bank

Key informant interviews were conducted with 21 study participants by an experienced Tanzanian researcher (JB) within the study team led the interviews, sometimes supported by the DS and RS (Table 3). Participant checking was employed throughout the interview for quality assurance. Interviews were conducted either face-to-face or remotely (via Zoom), depending on the respondents’ preferences. Interviews lasted, on average, 45 minutes. Interviews were conducted in a mix of Swahili and English, as participants working at this level felt most comfortable expressing themselves in this way. The study team held weekly reflexive meetings to refine lines of enquiry. Data were collected until saturation was reached (defined as no new information emerging from the respondents) [24].

**Table 3.** List of key informants and their affiliations.

Type of respondent	Contacted	Interviewed
*Government*	*12*	*9*
*Donors (WB, Bilateral, Multilaterals)*	*14*	*10*
*Other actors (CSOs)*	*2*	*2*
*Total*	*28*	*21*

### Data management

Data were held in Tanzania and analysed in country. Only soft copies were kept on password protected computers, and only anonymised summaries were shared with other co-authors.

### Positionality, trustworthiness, and transcription

The interviews were recorded and transcribed verbatim and then translated into English by JB. Transcripts were discussed as a team during regular reflexivity meetings to understand power and positionality within interviews and analysis and to refine lines of inquiry. GM is a senior author on the paper and holds an insider role as a GFF liaison officer for Tanzania, which helped identify key informants and relevant documents for review. However, GM did not participate in interviews or data coding to allow independent perspectives to emerge. DS and JB recognise their positionality as privileged Tanzanian’s working as health systems’ researchers and how this positionality as both insiders and outsiders to the policy space may have influenced the interviews and analysis. Furthermore, the broader GFF analysis collaboration (with teams from Uganda, Burkina Faso, Mozambique and Tanzania) met weekly for one hour from October 2022 to October 2023 to debrief on emerging themes and co-develop the coding framework.

### Data analysis

Data were analysed using thematic analysis [25] and themes were triangulated across KIIs and document reviews. Researchers familiarized themselves with transcripts and developed a codebook for further analysis with the broader GFF policy analysis collaborators. Interviews were coded in the QSR International NVivo 12 software based on deductive codes arising from the policy analysis triangle, to allow for comparison across the consortium contexts, and inductive codes emerging from the data [26]. Three team members (JB, RS, and DS) coded one transcript together to ensure that our interpretations were aligned. Data were then coded by JB to support the development of themes [27].

**Figure 1.**
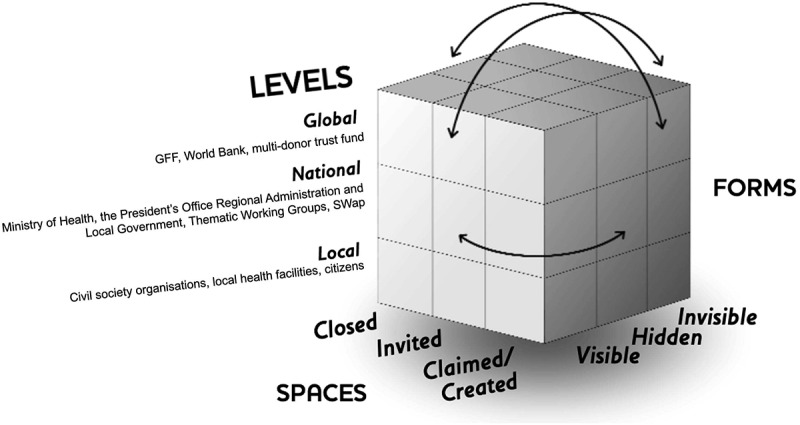
Adapted Gaventa power cube. This was adopted from [29].

Power emerged as central to our data so the conceptualization of power was further analyzed by applying Gaventa’s (2006) power cube (Figure 1). This theory argues that power must be understood in relation to how spaces for engagement are created (invited vs. claimed), forms of power (visible, hidden, and invisible), and the levels of power (from local to global) in which they occur [28,29]. We used the power cube to explore how power dynamics influenced GFF policy processes to support an understanding of policy processes over time. The data were mapped to the dimensions in Gaventa’s power cube [29] to understand the power structures within the processes of the GFF document development (Figure 1).

### Ethical considerations and validation

Ethical approval for this study was obtained from the Tanzania National Health Research Ethics Committee (NatHREC) ref. NIMR/HQ/R.8a/Vol.IX/3405, Ifakara Health Institute, Tanzania IHI/IRB/01–2021, and London School of Hygiene and Tropical Medicine. After being informed about the study, participants provide verbal or written informed consent to the researchers prior to the interviews. Participation was entirely voluntary. Confidentiality was maintained by removing any personal identifiers. Data files were password protected and stored in a secure location for five years following the study. All key informants provided voluntary, informed verbal or written consent to participate in the study.

We presented preliminary findings to research respondents, including MoH and MoF officials, RMNCAH-N implementors, CSOs, and the GFF secretariat, for feedback, which helped validate the findings with key stakeholders. The discussion, led by DS and JB, took place in November 2023, and was conducted both in-person and virtually with the participants. The purpose of these discussions was to validate the findings, providing valuable feedback that helped confirm them with key stakeholders

## Results

The results from the analysis of documents and interviews were synthesized and presented by the Walt and Gilson policy triangle domains, including context, content, actors, and process. We then applied an additional layer of policy analysis using the Gaventa Power Cube. The first and second waves of the GFF for both the ICs and PADs were considered.

### Policy context, content, actors and process

#### Policies and timeline

**Figure 2.**
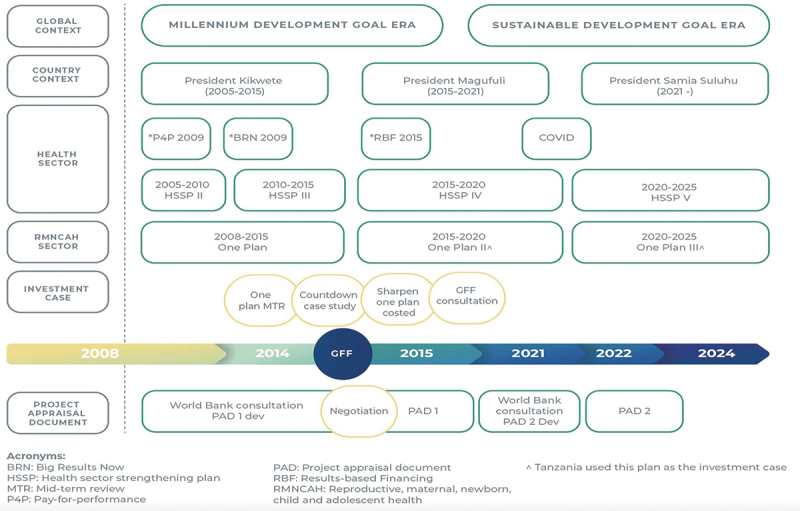
Timeline of Tanzanian health sector context and the GFF policy interaction (2008–2024).

Tanzania was one of the few countries to meet MDG4 for child survival but failed to meet MDG3 for maternal mortality or make progress for neonatal survival. The former president of Tanzania, Jakaya Mrisho Kikwete, launched the first ‘“Sharpened One Plan (2014–2015)”’ to accelerate the reduction of maternal, newborn, and child mortality in Tanzania, followed by the national RMNCAH-N Strategy One Plan II (2016–2020), with high-profile support at global and national levels. Figure 2 presents a timeline of key events linked to the GFF process, including the political environment, other national policy processes, and GFF-specific activities.

Four main GFF-related policy documents were identified for Tanzania, including two ICs and two PADs (Table 4). The ICs were the National Plans for RMNCAH-N, called the One Plan strategies (2016–2020 and 2021–2025).

**Table 4.** Summary of GFF documents in Tanzania in wave 1 (2016) and wave 2 (2021-2026).

Document	IC 1 (2016–2020)	PAD 1 (2015)	IC 2 (2021 –2026)	PAD 2 (2022)
*Source*	*One Plan II (2016–2020)*	*HSSP IV*	*One Plan III (2021 –2026)*	*HSSP V*
*Focus*	*SDGs*. *Interim targets: maternal mortality ratio from 432 to 292 per 100,000 live births, the neonatal mortality rate from 21 to 16 per 1,000 live births and under-five mortality rate from 54 to 40 per 1,000 live births*	*Primary health care*	*Maternal Mortality Ratio reduced from 250 per 100,000 live births to 100 per 100,000* *live births by 2025.Neonatal Mortality Rate reduced from 20 per 1,000 live births to 15 per 1,000 live births by 2025. Still birth rate reduced from 16 per 1,000 total births to 12 per 1,000 births by 2025. Under-Five Mortality Rate reduced from 50 per 1,000 live births to 38 per 1,000 live births by 2025.*	*Maternal and Child Health Investment Program*

The PAD serves to fund aspects of the IC; therefore, PAD 1 and PAD 2 were developed and linked to the National Health Sector Strategic Plans IV and V (2015–2020 and 2021–2026). PAD 1 focused on primary health care and preceded the IC by almost one year because of the World Bank’s linked programs. PAD 1 proposed results-based financing as the core mechanism for achieving impact (including scorecards at various levels of local government authorities) and described a programme estimated at US$2.62 billion or 55% of the Government’s health sector budget over five years. The GFF contribution to this programme was US$40 million. Eleven other development partners were expected to contribute US$290 million (or 11.1%) through parallel financing. The Government was to finance the remaining 77.5%. The PAD 2 was finalised in 2022 and focused on scaling up the provision and improving the quality of essential health care services, with a focus on maternal and child health. This investment comprised US$2.35 billion from the government, US$250 million from IDA credit, and US$25 million from the GFF Trust Fund. Both PADs apply the disbursement-linked indicators (DLIs) mechanism, which enables the prioritization of ‘tangible results’ and provides specific attention to health system implementation and multilevel change.

#### Actors

A high turnover meant that few actors were involved in both the IC and PAD, and varied actors were engaged in different short-term consultations. This turnover resulted in a slight disconnect between documents and was cited as a challenge. Often, however, actors would shift roles within the government or to other roles in the UN or the World Bank, meaning that the same pool of actors was sometimes involved in different positions across stages of the GFF. This meant that individuals often had oversight over several parts of the GFF, but few had a full picture. It was noted that there was space for multiple donors and actors at the table since ‘“*the needs are always bigger than the money”’ (KII, CSO #10).*

Actors also differed between IC and PAD processes, with the former being more inclusive and participatory. The One Plans were developed collaboratively with stakeholders, including the Ministry of Health (MoH), President’s Office Regional Administration and Local Government (PO-RALG), academic institutions, research organizations, civil society organizations, technical associations, development partners, UN agencies, and donor agencies. This is unlike the PAD 1 process, which only involved the World Bank team and key government ministries and was facilitated by a consultant who supported the initial phase of the GFF mechanism in Tanzania. This consultant played a role in the design, planning, and possibly the early implementation stages, bringing in specialized knowledge and experience. PAD 2 was more inclusive and engaged in structured mechanisms, such as Thematic Working Groups and SWAp.

#### Perceptions about the process

The use of existing national strategies and the loan mechanism from the World Bank (as opposed to private sector donations) was perceived positively, enabling a country-led model with the World Bank as ‘technical support’ for developing the PAD. The process was seen to ‘*compliment the government’s efforts*’ (KII, MoH #13) and bring people together.

The ICs in both phases in Tanzania were perceived as transparent and objective, utilizing a robust decision-making process. Key features included a common results framework, data sharing, and strengthened monitoring systems, with disbursement of funding to local health facilities, enabling power sharing to local health facilities.

… We received the funds, and then the health facility governing committee met to decide how to use the money. They review their needs-like fixing a broken toilet-seek quotes from local technicians, compare prices and quality, and choose the best option to get the work done. (KII, MoH #14)

Consensus on funding areas was achieved using diverse evidence sources such as routine data from the health information management system, data from national surveys, census data, case studies, and Mid-term Reviews. Mid-Term Reviews assessed the performance of the One Plan II (IC 1) implementation and informed stakeholders on progress, identified gaps, and helped inform priorities. For PAD 1, the Mid-Term Review recommended restructuring the Strengthening Primary Health Care for Results program by revising the results and DLI payment frameworks. It was highlighted that the prioritisation process was concentrated around measurable targets.

Oh, the evidence supported because there’s a lot of evidence to do many things, but we cannot do everything we would like to do, that’s been the problem in most places we have great national strategy. But they are not fully funded, so then we can all do something and we say we’re aligned to the national strategy, but it’s not linked to the available resources and that’s at the core of what [GFF are] trying to support countries in helping that prioritization. (KII, Donor #04)

This DLI mechanism, along with the prioritization process, was designed to enhance accountability by enabling the GFF and World Bank to monitor DLIs with the government’s results-based financing initiative. Respondents from the GFF and government felt more positively about this mechanism for achieving results and accountability as compared to the private sector or CSOs respondents, who were more critical, as illustrated in the quotes below. Overall, stakeholders acknowledged that a key advantage of the GFF mechanism was that funds were directly allocated to facilities, granting them autonomy over expenditure.

… because the government is the main implementer, the recipient of the money and the consumer of that money. It is not easy for it to evaluate itself, others who don’t receive money have to evaluate [it]. (KII, CSO #10)

there are about ten basket fund indicators, which we will monitor at all our health facilities in Tanzania, if they do well, they will be given money. … These indicators are at the level of RHMT (regional health management team), PO-RALG and the Ministry of Health because the Ministry of Health will go to supervise the RHMT to see that the RHMT are properly supervising the centres, etc. (KII, MoF #12)

### Power in policy processes

#### Places/levels of engagement:

The respondents noted how international policies and commitments ultimately shaped and informed the creation of national documents.

If we want to talk about relationship, … one of our functions was making sure that the national also is aligned to the international other policy and guidelines. At the end of the day when you look at the results, it also contributes to the global results. (KII, Donor #02)

The country-led model, disbursement of funds to the facility level, and autonomy of health facility governing committees were reported by some to be key strengths of the GFF mechanism and notable ways of sharing power among the global, national, and local levels. However, not all the respondents felt that this power was effectively shared. One respondent highlighted the conditions attached to the loan, highlighting the power held at the global level.

You know PAD is the World Bank tool. [Government] were not developing PAD. You have to distinguish this because that is a loan. … . It was decided and in fact it is a condition that you cannot access the GFF if you are not taking the loan. So, that was the negotiation. (KII, Donor #02)

It was also noted that there was an absence of participatory practice and citizen voice within the GFF process, which limited accountability.

What came out clearly is that the Government was hoarding the development process and operating it themselves, so even the process of revising One Plan II to make it to be investment case … reviewing the PAD document then, showing the direct link indicators which were used to indicate the priorities as far as the global financing facilities was concerned, all that was done by the Government. (KII, CSO #09)

it seems that the power is with the government and the bank [World Bank] and our interpretation is that the government is not the representative of the people, this is our interpretation because the government has a good goal but the mechanism to listen to the people is not there. So, he will do what he thinks is right, but we are asking the citizens, we are going to ask them, we are talking, and because the government is the main implementer, the recipient of the money, and the consumer of that money. It is not easy for it to evaluate itself; others who don’t receive money have to evaluate the way they look at things … . (KII, CSO #09)

The lack of engagement at the community level was also noted in response to shaping One Plan II, leading to a gap in thinking about RMNCAH-N beyond the facility and linking to a lack of invited spaces for participation:

I know we had to gather information from the community including the parents and the care takers. But, looking at how meaningful the engagement was and the extent of the engagement of the beneficiaries like parents and community members, possibly to gather their views and go further into the process of coming up with the national policy with the strategic document, it was somehow not so well funded … (KII, MoH #13)

In this way, although various stakeholders were involved across policy levels in different ways in the development of the two ICs and PADs, there remained concerns about how priorities were set, how, and by whom.

#### Spaces for engagement

As mentioned earlier, for the first round of funding, the World Bank consultants on behalf of the government developed the PAD. It was noted that where consultation was invited, largely through technical working groups, it was short-term, without feedback, and excluded some key stakeholders, such as members of parliament or youth. This sense of closed space or lack of meaningful engagement with communities, CSOs, NGOs, and the private sector was apparent and widely reported.

The World Bank gives money to the Ministry of Finance, and the money goes down. There is no place where you find private sector engagement; there is no place where you find private, not-for-profit engagement. (KII, CSO #09)

I am not sure what processes they went through until the IC was completed, but I know there was this kind of initial consultation, then … there was no continuity of participation from other stakeholders besides the initial consultation. (KII, International NGO #11)

It was also reported that the closed nature of decision-making spaces, even within the government, led to early confusion and misunderstanding of the role of the GFF as certain units, for example, family planning, adolescents were not involved at the outset.

Spaces for participation moved towards invited spaces in the second wave of the IC and PAD. This wave was noted to be more inclusive of stakeholders, such as thematic working groups, SWAp, the President’s Office – Regional Administration and Local Government (PO-RALG), and CSOs, who did not explicitly feature in the development of the first wave. However, these spaces did not always afford input opportunities. Despite being invited, participatory decision making was perceived by some to be limited by a lack of transparency.

President’s Office- Regional Administration and Local Government (PO-RALG) played a role to some extent, but its influence was limited because only a few people there had information about the GFF, leaving them somewhat behind. (KII, MoH #16)

CSOs also noted having to ‘claim’ their involvement rather than be invited in, which took time to materialize.’

An awakening call from international organizations prompted local CSOs to engage more meaningfully in the GFF processes. Two years later, we developed ways for CSOs to coordinate nationally and monitor GFF funding effectively, showing significant progress in their involvement … . Ultimately, the key point is that CSOs were engaged in the process quite late. (KII, CSO #09)

#### Forms of power

Visible power is exerted through decision-making structures and procedures. In the GFF, this relates to the process of funding allocation prioritization through PAD. Some respondents felt that the World Bank allocating funds to ‘measurable results’ was a clear expression of power:

… the World Bank had the mandate … to select which should be priority indicators to create the direct linked indicators, and the money comes from the indicators. So, they have the power to know or dictate the direct linked indicators. (KII, CSO #10)

So, World Bank comes in the negotiation of the loan. That is a funding modality. They have their measurements because it is performance based, they monitor performance and disbursement of resources… (KII, Donor #02)

However, respondents highlighted that the GFF and WB’s influence into what was funded skewed allocation towards renovation and construction of new hospitals, without the focus on human resources or infrastructure to make these fully functional.

I went to … a facility that has been renovated with GFF funds… They were struggling. … Not only shortage of staff, you can have shortage of equipment or reagents … issues connected with infrastructure, shortage of water or shortage of electricity, they can also influence the availability of services … like water, equipment or tools and the commodities and supplies. (KII, MoH #16)

Construction can be more political because everyone loves to see construction buildings whatever. But, how do you look into the aspect that can make this building function? That is where the challenge is… (KII, Donor #03)

Hidden power was exerted through decision making and agenda setting. This was highlighted above through the limited spaces for engagement from the community, private sector, and other critical players, meaning that agenda setting lacked these diverse perspectives. This means that certain considerations related to impact and sustainability are not adequately represented.

You know when it comes to RMNCAH … you can clearly see that [GFF] was not embracing all what it needs to be done or what needs to be prioritised … if you are focusing in the clinical side, and you are leaving out all what needs to be happening outside the clinical setting, we have the community-based interventions and we have the behavioural interventions where we need education targeting health care takers and adolescents and so forth. The circle is not complete and you will realise what is not expected to be realised. So, it is also a challenge. (KII, MoH #13)

Invisible power is enacted when inequities are normalized and accepted to the extent that certain groups are left out of policy considerations. This can be seen in the normalisation of measurable targets, but also with regards to vulnerable groups with specific health needs, such as people with disabilities, not being mentioned within the IC or included in decision-making or agenda setting. It was noted that a focus on priority groups was given more attention in One Plan III, yet this pertains only to women and youth, and disabled women for example are still absent.

Respondents noted that political sensitivities and the lack of cultural acceptance of family planning meant that Tanzania has not seen the same level of success as other countries. Therefore, key issues are sometimes not always explicit in documents.

I think the other thing, as you very well know [is the] political context in Tanzania is little sensitive around these issues. So, you have to carefully manage how to deal with these issues. Well, recognizing that they do need attention that sometimes happens without necessarily saying it in the documents. (KII, Donor #03)

Despite these concerns, explicit funding is allocated to family planning in PAD. Likewise, it was noted that recently, a small amount of funds has been allocated to adolescent health programming. Stillbirths, however, were not explicitly included in either PAD despite being in the IC. Respondents noted that stillbirth inclusion was implicit.

In the World Bank plus Basket funds, look holistically for the maternal, child and survival, so when we see these have been paid it means stillbirth is inclusive. (KII, International NGO #17)

We used to have nothing going into adolescent health programming … From zero to something though it has not increased a lot, but it is starting to put some resources. (KII, MoH #13)

## Discussion

This study is the first power analysis of GFF policy processes in Tanzania over time and includes the application of Gaventa’s power cube [30]. We described the GFF’s processes and mechanisms in synergizing financial resources to support national priorities in improving RMNCAH-N and then considered the power processes involved. Tanzania’s GFF mechanism presents a complex interplay of government leadership, stakeholder engagement, and the global policymaking environment surrounding women’s and children’s health. People and processes have changed across the two GFF rounds, exhibiting a degree of adaptive learning through a new global health initiative. The GFF have also since implemented various efforts to support adaptive learning, including annual reviews and via a country engagement platform [20]. The inclusivity of stakeholders varied, with early phases being less participatory than later stages. Visible power was evident in funding allocation prioritization by the World Bank, while hidden and invisible power was perceived by some to exclude community voices, private sector engagement, and vulnerable groups, such as people with disabilities. Autonomy at the local facility level improved accountability, yet gaps in addressing human resources and broader systemic needs persisted, underscoring the limitations in achieving holistic, sustainable health outcomes. Cultural and political sensitivities further shaped agenda-setting, particularly around family planning and adolescent health. The discussion considers how our findings on power link to participation, prioritization, inclusion, and differences across time.

### Power and participation

The GFF process respected the country’s own priorities by using One Plan II as the basis for the GFF IC, which was a real strength for country leadership. Despite this, power dynamics driving decision making were perceived to be held by the World Bank and selected groups within the Ministry of Health with limited transparency, notably in the first round. The relatively closed-door process also reportedly reduced the understanding of the GFF funding mechanism within the ministry. Spaces for participation are not neutral and their boundaries are shaped by power relations [14,30]. The spaces created for GFF policy development lacked the meaningful inclusion and participation of communities, vulnerable groups, the private sector, CSOs, and other key actors that could support accountability.

In particular, there were reports of CSOs claiming spaces for engagement with the GFF, following a call from international organizations. CSOs play crucial roles in delivering health services and advocacy but are not always actively involved in setting priorities or designing interventions [31]. This dynamic has also been reported in Uganda [32] and elsewhere in Tanzania [31]. Civil society organizations often have less formal power but can play a crucial role in ensuring that health programs are inclusive and responsive to community needs and that vulnerable groups are included. However, ensuring a fully representative process inclusive of community and CSO engagement in health financing decisions is not without its challenges and questioned by some as not necessarily needed for equity concerns to be included [33]. Nonetheless, broader engagement from CSOs can also mobilize community members to seek RMNCAH-N services and feel that they have been overlooked [15,34]. Just because they were not technical experts, did not mean they did not have relevant expertise. Indeed, as Salisbury *et al*. noted, CSOs play an especially important role in supporting adaptations to evidence-based interventions to improve outcomes funded through the GFF [35]. The GFF needs to continue adapting here to create platforms for dialogue and discussion with CSOs, and other community groups that represent underserved groups.

### Power and prioritisation

Data were perceived to be integral in prioritization, determining the most effective interventions, and proposing strategies expected to be feasible and impactful in Tanzania [22,36]. In both rounds in Tanzania, the ICs were seen as transparent and objective, with strong decision-making processes and a common results framework used to share data, strengthen monitoring, and disburse funds to local health facilities, demonstrating power sharing at the local level. Funding through the PAD and DLI mechanisms was seen to prioritize multiple needs. DLIs ensure that financial resources are released only when measurable progress has been made aimed at promoting accountability among implementing entities [37]. This is achieved through performance-based funding, including setting clear metrics and targets, and regular monitoring and evaluation [37]. However, it is also power-laden, with significant implications for controlling and allocating resources, and ultimately leaves the global level holding power, despite local-level committee monitoring. The normalization of measurable or visible targets as seen in global health is a further expression of invisible power, with the effect of excluding the other forms of targets that might have been prioritized by citizens, or CSOs. There is a challenge of multiple competing RMNCAH-N measurement frameworks and the GFF has a role in assessing these through participatory processes with CSOs, to ensure they speak to the priorities of citizens and streamlining indicators them for country application [20].

The GFF and World Bank, as major funders and technical advisors [2], were often perceived to have a substantial influence on the priorities and strategies outlined in the ICs and PADs. Their agendas and frameworks were also perceived to dominate the planning process, shaping the focus areas and resource allocation. Global health initiatives that leverage their financial and technical affluence to determine country priorities are not uncommon [38]. Global health metrics is political [20,39], in this case, other measurable health system components, such as human resources for health, were reportedly not prioritized by the GFF, limiting their impact and sustainability. The current GFF and UNICEF strategies have included approaches to human resources for health investments in Tanzania [40]. Balancing these dynamics is crucial for ensuring that the mechanism delivers equitable and effective health outcomes.

### Power and inclusion

Dominant ideologies shape perceptions of what is acceptable through the exercise of ‘invisible power’ (Gaventa, 2006) [8,30]. Invisible power was observed in how GFF policy documents did not fully engage in issues of equity, particularly with regard to the inclusion of vulnerable populations or marginalized groups. Policy processes also had a narrow agenda that focused on facilities but did not include the needs of the communities, reflecting a biased agenda and a form of hidden power that played out in the GFF. This risks the invisible remaining as such – systematic discrimination and marginalization may limit the awareness of systems of oppression (recognition) as well as the political voice and agency required to meaningfully participate in shaping agendas in ‘invited spaces’ created by the governments and the GFF [8,10,13].

Stillbirths were also not explicitly given attention and were said to be implicit within other funding structures. This is despite global calls [20,39]. Challenges to invisible power may require more attention to specific issues, such as stillbirth, which is also a bottleneck in attaining SDGs [41,42]. Other studies reported that groups not prioritized in ICs or PAD received little or no funding [41], yet stillbirths were included within IC 2. Adolescents’ health has also been reported to receive minimal funding. A review of GFF policy documents, including Tanzania’s by George *et al*. [43] shows that adolescents were mentioned with regard to issues such as adolescent fertility and early marriage rather than as an opportunity. This narrow scope may hinder the prioritization of health needs and challenges faced by adolescents in Tanzania [44]. The GFF therefore needs to reevaluate funding lines to ensure that all groups are catered for to ensure this focus does not yet lost.

### Differences across time

The closed consultant-driven approach in PAD 1 contrasts with the more inclusive mechanisms used in PAD 2, reflecting a possible evolution in the GFF’s strategy towards more collaborative and integrated planning and implementation processes. There were more engagement platforms (invited spaces) in the second PAD, supporting a diverse range of voices and perspectives in decision-making, enhancing the legitimacy and effectiveness of the GFF mechanism [2]. This multi-sectoral approach is critical for robust policymaking [5,35]. Despite this, respondents still felt that there was a limited follow-up process after engagement and limited participatory priority-setting processes. Feedback loops are crucial for adaptive management [16,45] and broader accountability structures beyond the government, as highlighted by respondents. Therefore, this requires an examination of future phases and continued adaptive learning approaches [20].

### Policy implication

The Tanzania case highlights that while the GFF’s country-led model can align with national strategies, meaningful inclusion of civil society, communities, and marginalized groups has further room to improve building on progress made. Policymakers should invest in strengthening inclusive engagement platforms – especially for underserved groups – throughout the investment case and PAD development, ensuring transparency and feedback loops. Safeguards are needed to ensure domestic priorities are not overshadowed by donor agendas, while embedding adaptive learning across funding phases to strengthen ownership, accountability, and equity in RMNCAH-N outcomes. Future policy design should also balance performance-based financing with investments in systems components to deliver on longer term goals. They should also explicitly include targets and funding lines for vulnerable groups and high burden issues, such as stillbirth and adolescents.

### Strength and limitations

Our analyses draw on a range of documents and respondents and are triangulated across these data sources. A multidisciplinary team also brings about varied positionalities. The GM position as an ‘insider’ may also have introduced potential bias, but we used reflexivity sessions to mitigate this and ensured that GM was not involved in the analysis and data collection. The team within the broader collaboration was also a strength; we met frequency to facilitate knowledge exchange and learning across contexts. The use of the Gaventa Cube was helpful in our analysis, but was not explicitly used to frame discussions in interviews, which is a limitation, although issues of power and relational dynamics were explored. Interviews were conducted retrospectively on the processes that have occurred over the past eight years. While being retrospective allowed for more direct reflections on power, we asked about historic events that may have been subject to recall bias. The movement of respondents between the government and the GFF was common across the two waves of the GFF. Therefore, some perspectives may not have been included, although this did mean that respondents spoke from varied positionalities. Other perspectives were also not included in this research; for example, we did not discuss affected communities.

## Conclusions

The GFF leveraged national health strategies and data-driven prioritization to align IC and PAD with RMNCAH-N goals and supported funding with measurable outcomes through DLIs. Decision-making processes involve government authorities holding visible power, driving the strategic direction of health programs, and financial planning. The World Bank and its development partners also wielded substantial influence at the global level because of their financial contributions and technical expertise. The GFF process is designed to be inclusive, yet we found that reality can fall short, with civil society engagement being low and often powerless. The GFF has the potential to create a balanced and effective framework for improving RMNCAH-N in Tanzania, but explicit attention is needed to meaningfully engage with a broader range of stakeholders, particularly citizens for whom the GFF is meant to serve.

## Supplementary Material

COREQ Checklist_GFF_TZ.pdf

## Data Availability

The anonymized dataset used and/or analyzed in this study may be available from the corresponding author on reasonable request. This paper is part of a *Global Health Action* Special Series. A more detailed explanation of the full study and sub-analyses will be available in Volume 17–01.
